# Evidence for an intrinsic factor promoting landscape genetic divergence in Madagascan leaf-litter frogs

**DOI:** 10.3389/fgene.2015.00155

**Published:** 2015-05-15

**Authors:** Katharina C. Wollenberg Valero

**Affiliations:** Department of Natural Sciences, College of Science, Engineering and Mathematics, Bethune-Cookman UniversityDaytona Beach, FL, USA

**Keywords:** landscape divergence, speciation, riverine barriers, topographical complexity, Madagascar

## Abstract

The endemic Malagasy frog radiations are an ideal model system to study patterns and processes of speciation in amphibians. Large-scale diversity patterns of these frogs, together with other endemic animal radiations, led to the postulation of new and the application of known hypotheses of species diversification causing diversity patterns in this biodiversity hotspot. Both extrinsic and intrinsic factors have been studied in a comparative framework, with extrinsic factors usually being related to the physical environment (landscape, climate, river catchments, mountain chains), and intrinsic factors being clade-specific traits or constraints (reproduction, ecology, morphology, physiology). Despite some general patterns emerging from such large-scale comparative analyses, it became clear that the mechanism of diversification in Madagascar may vary among clades, and may be a multifactorial process. In this contribution, I test for intrinsic factors promoting population-level divergence within a clade of terrestrial, diurnal leaf-litter frogs (genus *Gephyromantis*) that has previously been shown to diversify according to extrinsic factors. Landscape genetic analyses of the microendemic species *Gephyromantis enki* and its widely distributed, larger sister species *Gephyromantis boulengeri* over a rugged landscape in the Ranomafana area shows that genetic variance of the smaller species cannot be explained by landscape resistance alone. Both topographic and riverine barriers are found to be important in generating this divergence. This case study yields additional evidence for the probable importance of body size in lineage diversification.

## Introduction

Mechanisms of lineage diversification are still poorly understood biological phenomena. Large animal radiations are thought to be the result of the complex interaction between parameters of past and present physical environments (extrinsic factors), and factors intrinsic to the organisms (e.g., aspects of the phenotype and its evolutionary history). In order to obtain a quantitative understanding of the process of speciation, the relative importance of both types of factors needs to be assessed. For example, in African cichlid fishes, if optimal values for both extrinsic and intrinsic factors are in concordance (e.g., solar radiation and lake depth as extrinsic and sexual dichromatism as intrinsic), the likelihood of lineage diversification can be partially predicted (Wagner et al., 2012). The endemic Malagasy frog radiations have been extensively studied for their phylogenetic relationships (e.g., Wollenberg et al., 2007, 2008, 2011; Vieites et al., 2009) and biogeography, while less is known about their ecology (except for general ecological modes like habitat and breeding biology, Glaw and Vences, 2007). These frogs are sharing the island with other endemic radiations (Lemurs, Tenrecs, Vanga birds), resulting in patterns of diversification being shared among radiations and Madagascar thus constituting a good model region to infer the processes causing species diversity, species richness and endemism (Wollenberg et al., 2008; Vences et al., 2009). Regarding the Malagasy frogs, both extrinsic and intrinsic factors have been studied, with extrinsic factors usually being related to the physical environment (landscape, climate, river catchments, mountain chains), and intrinsic factors being clade-specific traits or constraints (reproduction, ecology, morphology, physiology). Additionally, geographic range size can be the result of and act as an extrinsic as well as an intrinsic factor (reviewed in Cooper and Purvis, 2009).

Most research in Madagascan frogs has been conducted on extrinsic factors, as the available data on genetics and distribution data facilitates this type of study. Following general practice in biogeographic inference, observed patterns (e.g., two phylogenetic clades being situated in two different climatic regimes), are being related to the diversification process (the difference in climate led to the evolution of the two groups). However, despite that extrinsic factors often correspond to phylogeographic splits of more basal clades (e.g., Kaffenberger et al., 2012) these large-scale extrinsic factors fail to explain the majority of the more recent speciation events. In the case of the Madagascan frog genus *Gephyromantis*, three basal splits in the phylogeny mirror distribution areas in three different areas of faunal endemism, but most of species diversification events (46 in total, not considering taxonomic uncertainty or possible extinctions), could not be explained by such barriers (Kaffenberger et al., 2012). To this end, more fine-scale factors impeding gene flow over the landscape need to be studied. Given the fact that most Madagascan amphibian species diversity is located in the Eastern Rainforest biome corresponding to an underlying escarpment, topographic heterogeneity might be an important factor contributing to this diversity (Wollenberg et al., 2008). For example, Guarnizo and Cannatella (2013) found that elevational bands are the most important predictor for diversification between recent sister species of Andean *Dendropsophus* frogs. Other studies have emphasized the importance for smaller rivers, or montane ridges as barriers for frog dispersal (e.g., Zhan et al., 2009; Gehring et al., 2012). As for intrinsic factors, recent studies in frogs have emphasized the importance of body size on clade diversity (Van Bocxlaer et al., 2010; Zimkus et al., 2012). Testing this hypothesis for the largest Malagasy frog radiation (Mantellidae, with 242 species), revealed that smaller species indeed have higher clade diversity, smaller distribution areas, and higher mitochondrial substitution rates (Wollenberg et al., 2011). However, this trend was not statistically significant within the mantellid frog radiation (Wollenberg et al., 2011), potentially due to the small portion of large frogs with large range sizes within mantellids available for comparative testing. Pabijan et al. (2012) found nucleotide divergence between spatially separated populations in a subset of mantellid frogs to be inversely correlated with body size, which supports the hypothesis that body size as an intrinsic factors plays a role in generating genetic diversity.

Without doubt, both intrinsic and extrinsic factors contribute to generating Madagascan amphibian species diversity (Vences et al., 2009; Brown et al., 2014). The question is, what is their relative contribution? Under the assumption that similar processes of selection will produce similar outcomes, one way to test such interactions is to compare patterns across sister species that only differ in intrinsic factors. The subgenus *Gephyromantis* (Mantellidae/*Gephyromantis*) is a group of diurnal, inconspicuous leaf-litter frogs endemic to Madagascar. From what is known, many of the up to 18 species of the subgenus deposit eggs on land, and have pseudo-direct development, with varying degrees of reduction of a free-swimming tadpole stage (Randrianiaina et al., 2011). Within the subgenus *Gephyromantis*, one monophyletic lineage is comprised of small, microendemic frogs (containing the species *Gephyromantis enki*, *G. blanci*, *G. runewsweeki*) and a monophyletic lineage of larger frogs with wider distribution (containing populations of the species *Gephyromantis boulengeri*). While *G. runewsweeki* and *G. blanci* are elusive and probably only occurring in single, small patches of habitat, *G. enki* is widely distributed in Ranomafana National Park (RNP). There, it inhabits mid- to high-elevations. *G. boulengeri* occurs from RNP to Nosy Mangabe in the North–East, and is also widely distributed in lowlands. Since these two lineages containing small and medium sized frogs are sister to each other, (1) they are of the same evolutionary age (Wollenberg et al., 2011). (2) They share the same general mode of reproduction, thus being similar in breeding biology. (3) Both clades being diurnal and occupying similar calling positions, they are ecologically similar. (4) Inhabiting partly the same area (Ranomafana) means, that there they are faced with the same obstacles to dispersal. The main differences observed between *G. enki* and *G. boulengeri* are (1) body size, and (2) range size. Because of their similarities in most other life-history traits relevant for amphibians, these two species therefore comprise an ideal system to test whether different body and range size cause different patterns of genetic divergence over a landscape. In this paper I test whether population genetic structure of these two species is affected by landscape resistance and geographical barriers the same way or differently. Within RNP, both species occur on both sides of a large river (the Namorona River). Further, elevation steadily increases within a short distance. Wollenberg et al. (2011) proposed that a microendemic phenotype (small frogs with small range sizes) would diversify faster than frogs with a combination of larger range and body size. This leads to the expectations of: (1) Increased level of genetic differentiation in *G. enki* compared to *G. boulengeri*, and (2) Topographic structures such as elevational bands or the Namorona River constituting strong barriers to diversification for *G. enki*.

## Materials and Methods

To test these hypotheses, I analyzed sequences of the mitochondrial cytochrome b (*cytb*) gene and the nuclear recombination-activating gene 1 (RAG1) gene of populations of both clades and other members of the subgenus *Gephyromantis*. For *cytb*, 106 sequences of *G. enki* and 58 sequences of *G. boulengeri* were analyzed. For *RAG1*, 30 sequences of *G. enki* and 33 sequences of *G. boulengeri* were analyzed. Amplification and sequencing protocols for newly determined sequences follow Kaffenberger et al. (2012). Specimen and locality information, and Genbank accession numbers are listed in Supplementary Table S1. For visualization of genealogical relationships, reticulate evolutionary networks were constructed from each (phased for RAG1) alignment with the software NETWORK V.4.611 (Fluxus Technology Ltd, 1999–2012). The Median-joining algorithm was applied (Bandelt et al., 1999). The resulting networks were edited in NETWORK and Corel Draw (V.X6). Extensive networks were constructed for all haplotypes without removal of single sequence haplotypes.

Locality datasets were constructed for both species (for coordinates, see Supplementary Table S1) as input files for the spatial analyses. First, I constructed environmental niche models for both *G. enki* and *G. boulengeri* in the software Maxent 3.3.3k under standard settings (Phillips et al., 2006; Phillips and Dudík, 2008). The models (random seed) were created per species for Madagascar as background with 10,000 background points. A resistance map was then calculated for each species by applying the circuit theory to the Maxent models (software Circuitscape V. 4.0, McRae, 2006; McRae and Shah, 2009). In this approach, landscapes are represented as conductive surfaces, with high resistances assigned to barriers for movement and dispersal (McRae and Shah, 2009). Output was set to resistances. These resistance maps are commonly used to predict patterns of gene flow. Values for landscape resistance and for elevation were extracted from the resistance map and a digital elevation model for each sampling locality per species in DIVA GIS (V.7.5.0, Hijmans et al., 2001).

Genetic distance matrices of *G. enki* and *G. boulengeri* were constructed in MEGA (V.6, Tamura et al., 2004, 2013) using the Maximum Composite Likelihood model. All codon positions were included. The genetic distance matrices were spatially decomposed using the PCNM function (Principal Components of the Neighborhood matrix, Borcard and Legendre, 2002; Borcard et al., 2004) in R (package vegan, Oksanen et al., 2011). PCNMs with negative Eigenvectors or very small values were then discarded prior to analysis.

One dataset per species containing the genetic distance PCNMs and the extracted values for elevation and landscape resistance was assembled for statistical analysis (StatSoft, Tulsa, OK, USA). A regression analysis was conducted with landscape resistance and elevation as independent variables and the genetic PCNMs as dependent variables, in order to compute residuals. These residuals represent the remainder of the genetic variance of each species and marker, after removing the effect of isolation by resistance and topography. Two data points of *G. boulengeri* were removed from the cytb dataset, as their residuals exceeded twice the size of the standard deviation and thus represented outliers. The regression was then repeated with exclusion of these two data points. Localities included were (1) within RNP: Ranomafana, Station Valbio, Valbio: Campsite, Ambatolahy, Sahamalaotra, Kidonavo, Ranomafanakely, Sakaroa, Talatakely II, Talatakely II, Talatakely III, and Station Thermale, and (2) outside RNP: Ifanadiana, Ambohitsara, Andasibe (Supplementary Table S1). To determine whether the remainder of genetic variance differs between the smaller species and the larger one, a Kruskal–Wallis test was then performed in STATISTICA.

To analyze the genetic divergence of the smaller species *G. enki* within RNP, which represents the extent of its spatial distribution, a spatial representation of barriers to dispersal was computed using the methods of Manniet et al. (2004) within the software Barrier 2.2. Barrier identifies spatial boundaries corresponding to areas of high genetic distance using Monmonier’s maximum difference algorithm (Manniet et al., 2004). These barriers were computed for *cytb* and *RAG1* separately. Arlequin (V. 3.5, Excoffier and Lischer, 2010) was used to assess haplotype diversity of sampling localities, and to test hypotheses of diversification across barriers for each genetic marker separately. For this purpose, analysis of molecular variance (AMOVA) was run on two groups; (1) including populations on both sides of the Namorona river, and (2) including populations separated by elevational bands. Populations were grouped according to north and south of the Namorona River, with a northern group containing Ranomafana, Valbio, Valbio: Campsite, Ambatolahy, Sahamalaotra, Kidonavo, Ranomafanakely, and a southern group containing Sakaroa, Station Thermale, Talatakely I, Talatakely II, and Talatakely III sampling localities. Populations grouped by three elevational bands were 1-Ranomafana, Station Thermale (630–640 m.asl.), (2) Ambatolahy, Campsite, Station Valbio, Talatakely I, Talatakely II, Talatakely III, Sakaroa (900–1000 m.asl.), (3) Sahamalaotra, Ranomafanakely, Kidonavo (1140–1160 m.asl.). AMOVAs were performed using pairwise differences and 10,000 random permutations. Significance of recovered fractions was tested with 10100 random permutations.

## Results and Discussion

### Genetic Diversity of the Smaller Species *G. enki* within RNP is Greater than in the Larger Species *G. boulengeri*

**FIGURE 1:**
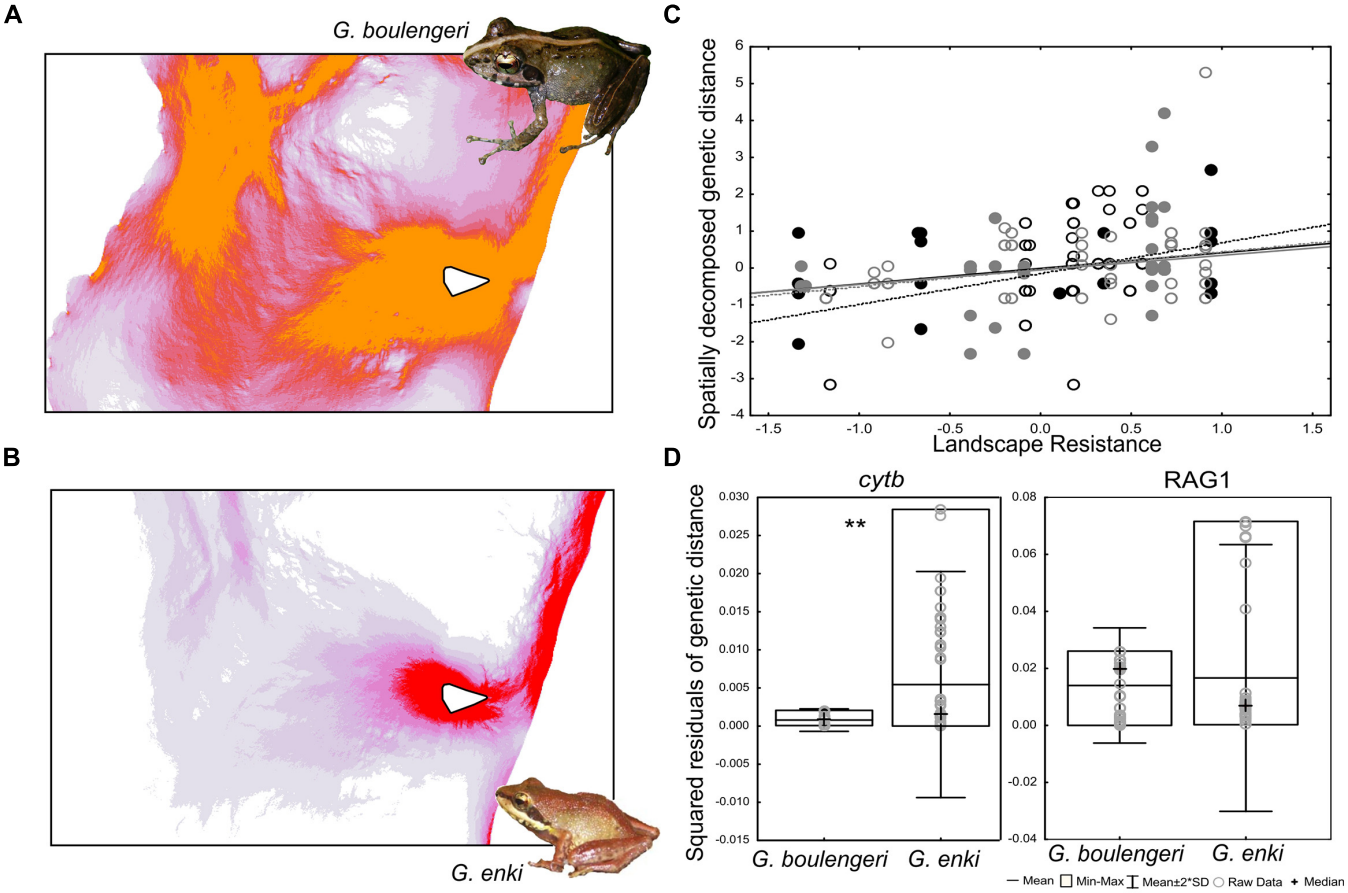
Landscape genetic divergence of two *Gephyromantis* species, corrected by isolation-by-resistance. **(A,B)** Show resistance maps computed for each species and images of the frogs. Dark red and orange show high landscape connectivity, respectively. White triangle represents a minimum convex polygon of the sampling localities within Ranomafana National Park (RNP). **(C)** Shows a significant but weak correlation between spatially decomposed genetic distances and landscape resistance (gray – RAG1, black – *cytb,* solid circles and solid fit line – *G. boulengeri*, open circles and dotted fit line – *G. enki*). **(D)** Squared residuals of genetic distance to landscape resistance compared between the two species. Double asterisks denote significance of differences computed with Kruskal–Wallis test. *G. enki* in average has a higher residual variance in genetic distance over its distribution area than *G. boulengeri*.

Maxent returned good AUC values for both *G. enki* (0.99) and *G. boulengeri* (0.99) for the environmental niche model computation. The resistance maps computed on the basis of these environmental niche Models showed that landscape resistance for both *G. enki* and *G. boulengeri* is low in the Ranomafana area (**Figure 1**). Regression results reveal that some genetic differentiation of both *G. enki* and *G. boulengeri* can be explained by landscape resistance and the prevalence of different elevational bands in the area (isolation-by-resistance, McRae, 2006). A correlation between landscape resistance and the spatially decomposed genetic distances is shown in **Figure 1**. This analysis included all populations for both species. The regression models were significant for the *G. enki cytb* (*R*^2^ = 0.45, *p* < 0.0001) and the *G. boulengeri* RAG1 (*R*^2^ = 0.1, *p* < 0.04) datasets, but not for the *G. enki* RAG1 (*R*^2^ = 0.03, *p* < 0.4) and the *G. boulengeri cytb* (*R*^2^ = 0.03, *p* < 0.5) datasets. Residuals were then computed and used for hypothesis testing. The smaller species *G. enki* in average showed higher residual genetic variance than the larger species *G. boulengeri* after controlling for landscape resistance and topography. A Kruskal–Wallis test for landscape-independent genetic divergence was significant for cytb, but not for RAG1 [KW-H: 11.88; *p* = 0.0006 for both markers combined (not shown), KW-H: 7.1322, *p* = 0.0076 for cytb alone, KW-H: 0.5336, *p* = 0.4651 for RAG1 alone; **Figure 1**].

Conclusively, the results confirm the expectation that among two ecologically similar sister species of frogs, the smaller species shows higher genetic variance over the same geographic area, independently from isolation-by-resistance. These results correspond well to the analysis of Pabijan et al. (2012), who found a similar trend for a set of mantellid frogs over a larger distance (between Andasibe and RNP).

### Landscape Effects on Diversification of the Smaller Species *G. enki*

**FIGURE 2:**
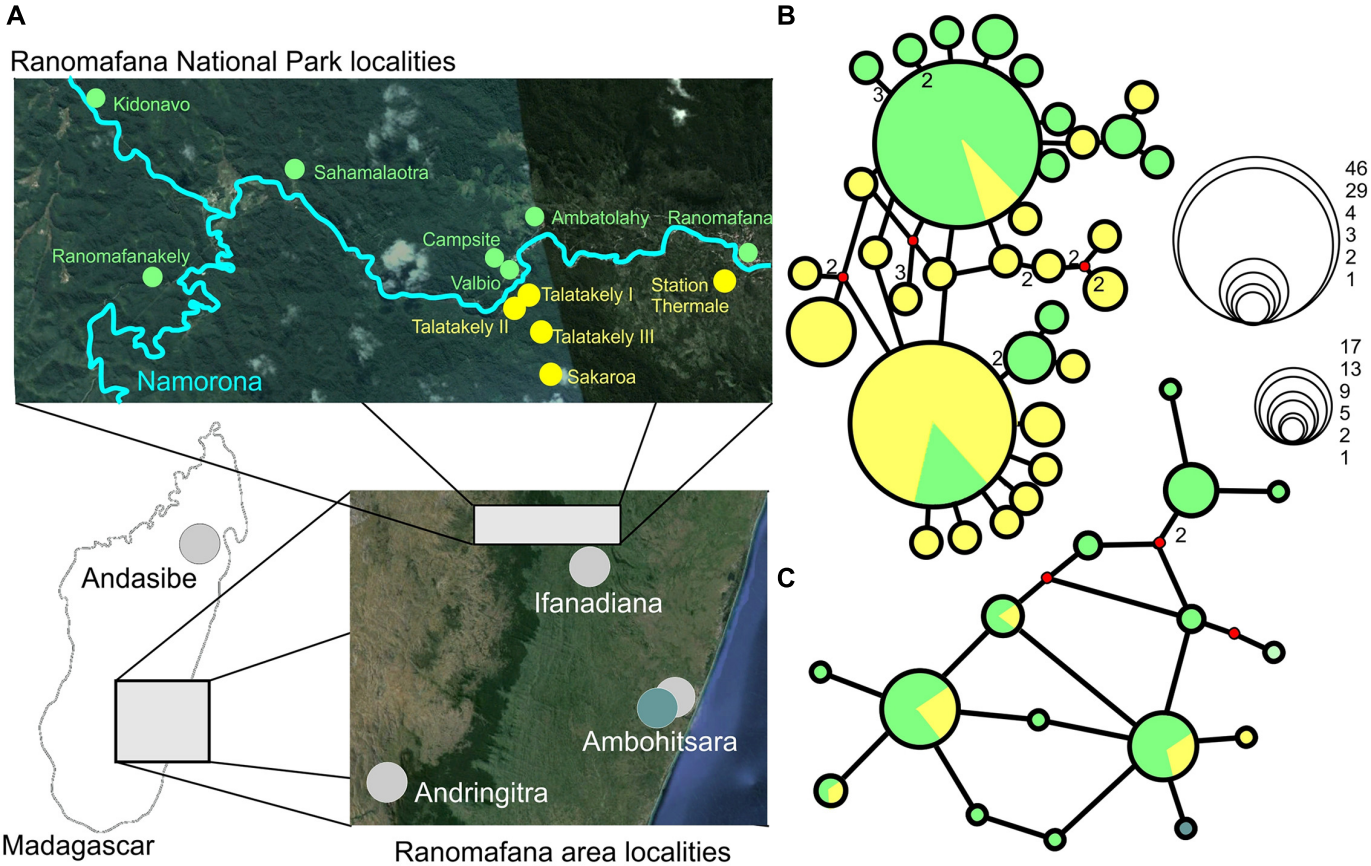
**(A)** Sampling localities, and Median joining haplotype networks of *G. enki* haplotypes found inside RNP. **(B)** *cytb*, **(C)** *RAG1*. Green – sampling localities north of Namorona River/RNP, yellow – sampling localities south of Namorona River/RNP. Teal – Ambohitsara. Red dots on networks denote median vectors, numbers represent number of mutational steps (default = 1). Bubble sizes correspond to number of sequences per haplotype as shown in the scale inset figure (Map Data: Google, 2015 CNES/Astrium, Image Landsat, 2015 Digital Globe).

**FIGURE 3:**
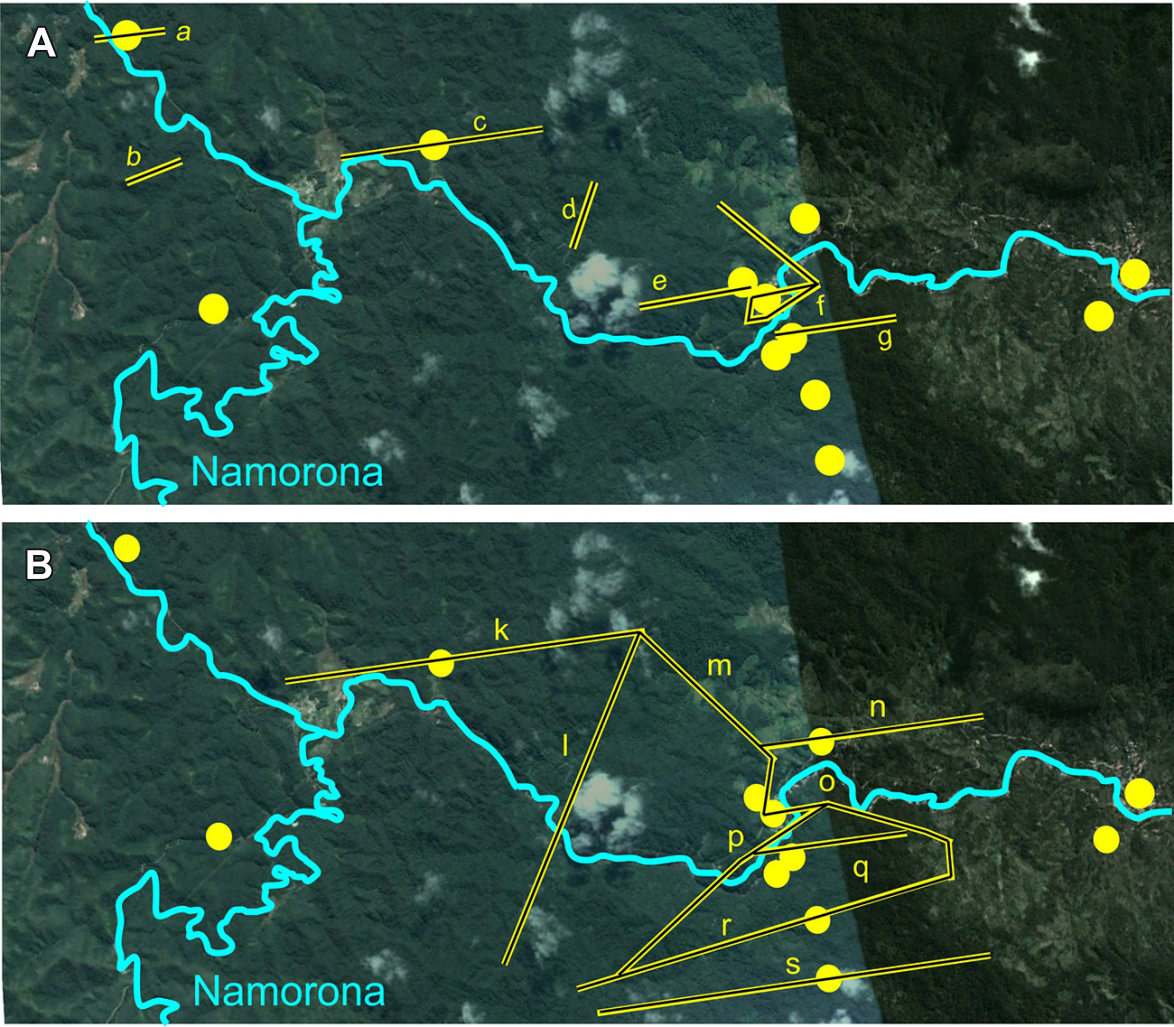
Results from the BARRIER analysis showing barriers to dispersal within RNP. Sampling localities of *G. enki* in yellow. **(A)** Based on *cytb* Maximum Composite Likelihood distances; a-g- *G. enki* dispersal barriers as yellow bars. **(B)** based on *RAG1* Maximum Composite Likelihood distances; k-s- *G. enki* dispersal barriers as yellow bars; Map Data: Google, 2015 Digital Globe.

Haplotype networks generated for *G. enki* showed a separation of haplotypes between localities north and south of the Namorona River (**Figure 2**). While the RAG1 network showed some haplotypes restricted to the northern populations, the southern populations were all allocated to haplotypes that also occurred north of the Namorona River. The faster evolving cytb gene, however, showed a clear distinction between two haplotype groups that differed in one mutated position between northern and southern banks of the river (**Figure 2**). This distinction was not perfect, but hints at the Namorona River being a barrier for these frogs. The estimated dispersal barriers for *G. enki* exist this riverine barrier, close to and parallel to the Namorona River (e.g., a,c,e,f,g, **Figure 3**). Additionally, barriers perpendicular or far away from the Namorona River (e.g., b,d, **Figure 3**) suggest the importance of elevational bands for impeding *G. enki* gene flow. The AMOVA for two classes of barriers (riverine versus elevational) confirmed that *G. enki* showed both significant within-population differentiation as also high within-group differentiation (**Table 1**). Furthermore, significant among-group variation was detected for both classes of barriers in *cytb*, but not in *RAG1*.

**Table 1:** Analysis of molecular variance (AMOVA) for the partitioning of genetic variation of the mitochondrial *cob* gene and the nuclear RAG1 gene within and among populations of G. enki.

	Opposite sides of river	Across elevational bands
***cytb***
Among groups	32.04**	**19.22**
Among populations within groups	13.57**	22.88**
Within populations	54.39**	57.89**
***RAG1***
Among groups	-1.37	6.22
Among populations within groups	25.68**	20.80*
Within populations	75.68**	72.97**

Shown is percentage of variance explained for: populations grouped according to position relative to the Namorona River, and populations grouped by elevational bands. Significance of fractions (covariance components) tested with 10100 permutations, indicated with *p < 0.05 or **p < 0.01. Significant among-group divergence in bold.

Sampling localities on opposite sides of the Namorona River explained 32.04% of the molecular variance of *cytb* found in *G. enki*. The location of populations on either side of the Namorona River was a significant predictor for genetic divergence, also the elevational bands (perpendicular to the Namorona River) explained a significant portion of molecular variance in *cytb* of *G. enki*. With 19.22%, this grouping explained 12.82% less variance than the riverine barrier grouping. No single best predictor for genetic divergence of *G. enki* was found, which indicates that any topographic structure can act as a barrier for a small frog, not only large rivers.

The Namorona River is therefore a stronger barrier to dispersal of *G. enki* than the elevational profile of RNP. This might be explicable by the fact that the subgenus *Gephyromantis* is the only clade of Malagasy frogs that has a terrestrial mode of development. Tadpoles of many Malagasy frog species are adapted to fast-flowing streams and can therefore be expected to cross a riverine barrier, but not *G. enki* (Glaw and Vences, 2007; Randrianiaina et al., 2011). These results conform to the expectation that fine-scale topography, in this case located in the lower montane RNP in Madagascar, contains multiple barriers for diversification for a small species of frog which are not limiting gene flow for its larger sister species. Besides the classic question of whether large scale biogeographic barriers such as the Amazon impedes dispersal and gene flow (Lougheed et al., 1999; Gascon et al., 2000, 2006), recently also smaller water bodies have been confirmed as barrier for recent amphibian diversification events (Ratsoavina et al., 2013; Munoz-Ortiz et al., 2014; van de Vliet et al., 2014, but see Dahl et al., 2013).

In addition to the confirmation of a small river and fine-scale topography serving as a dispersal barrier for a small rainforest frog, this study also confirms the hypotheses that a small and a larger sized, ecologically similar sister species pair of frogs show different patterns of landscape divergence. Adding to recent evidence for an effect of life-history traits on evolutionary processes shaping biodiversity (Fouquet et al., 2012), this case study shows that intrinsic factors such as body size, and associated distribution area size, might be important for diversification of Malagasy frogs.

## Conflict of Interest Statement

The Reviewer Miguel Vences, declares that, despite having collaborated with author Katharina C. Wollenberg Valero, the review process was handled objectively. The author declares that the research was conducted in the absence of any commercial or financial relationships that could be construed as a potential conflict of interest.
