# Supplementary material for: Evidence for an intrinsic factor promoting landscape genetic divergence in Madagascan leaf-litter frogs
Source: Front Genet. 2015 May 15;6:155. doi: 10.3389/fgene.2015.00155 (PMC4470402; doi:10.3389/fgene.2015.00155)
Supplement: Supplementary file 1 [file Table_1.DOC]

**Supplementary Material**

To the manuscript

Evidence for an intrinsic factor promoting landscape genetic divergence in Madagascan leaf-litter frogs

By Katharina C. Wollenberg Valero

**Contains: 1 Supplementary Table**

**Supplementary Table 1.** Species studied, collection number of voucher specimens and their collecting localities, and Genbank accession numbers of sequences obtained. ZCMV refers to field numbers of Miguel Vences, Braunschweig, Germany.

| **Species** | **Voucher number (ZCMV-number)** | **Sampling locality** | **Longitude** | **Latitude** | **GenBank Accession No. (cytb)** | **GenBank Accession No. (RAG1)** |
| --- | --- | --- | --- | --- | --- | --- |
| *G. enki* | 5129 | Talatakely II | -21.2580 | 47.4220 | KR376054 |  |
| *G. enki* | 5204 | Ranomafanakely | -21.4891 | 47.4891 | KR375993 |  |
| *G. enki* | 5205 | Ambatolahy | -21.2485 | 47.4272 | KR375973 | KR537839 |
| *G. enki* | 5206 | Ambatolahy | -21.2485 | 47.4272 | KR375974 |  |
| *G. enki* | 5207 | Ambatolahy | -21.2485 | 47.4272 | KR375975 |  |
| *G. enki* | 5208 | Talatakely II | -21.2580 | 47.4220 | KR376057 | KR537861 |
| *G. enki* | 5209 | Talatakely II | -21.2580 | 47.4220 | KR376058 | KR537862 |
| *G. enki* | 5212 | Ambatolahy | -21.2485 | 47.4272 | KR375976 |  |
| *G. enki* | 5213 | Ambatolahy | -21.2485 | 47.4272 | KR375977 |  |
| *G. enki* | 5214 | Ambatolahy | -21.2485 | 47.4272 | KR375969 |  |
| *G. enki* | 5215 | Ambatolahy | -21.2485 | 47.4272 | KR375970 |  |
| *G. enki* | 5217 | Talatakely II | -21.2580 | 47.4220 | KR376055 | KR537863 |
| *G. enki* | 5218 | Talatakely II | -21.2580 | 47.4220 | KR376056 |  |
| *G. enki* | 5220 | Station Valbio: Campsite | -21.3163 | 47.4766 |  | KR537867 |
| *G. enki* | 5221 | Station Valbio: Campsite | -21.3163 | 47.4766 |  | KR537857 |
| *G. boulengeri* | 5237 | Ambohitsara/Tsitola | -21.3574 | 47.8154 | KR376098 |  |
| *G. enki* | 5241 | Sahamalaotra | -21.3541 | 47.5691 | KR376002 | KR537847 |
| *G. enki* | 5242 | Sahamalaotra | -21.3541 | 47.5691 | KR376003 | KR537848 |
| *G. enki* | 5243 | Sahamalaotra | -21.3541 | 47.5691 | KR376004 | KR537849 |
| *G. enki* | 5246 | Sahamalaotra | -21.3541 | 47.5691 | KR376006 |  |
| *G. enki* | 5248 | Sahamalaotra | -21.3541 | 47.5691 | KR376007 |  |
| *G. enki* | 5249 | Sahamalaotra | -21.3541 | 47.5691 | KR376008 |  |
| *G. enki* | 5250 | Sahamalaotra | -21.3541 | 47.5691 | KR376009 |  |
| *G. enki* | 5251 | Sahamalaotra | -21.3541 | 47.5691 | KR376010 |  |
| *G. enki* | 5252 | Sahamalaotra | -21.3541 | 47.5691 | KR376011 |  |
| *G. enki* | 5254 | Sahamalaotra | -21.3541 | 47.5691 | KR376005 |  |
| *G. enki* | 5256 | Sahamalaotra | -21.3541 | 47.5691 | KR376012 |  |
| *G. enki* | 5257 | Talatakely III | -21.2625 | 47.4246 | KR376069 |  |
| *G. enki* | 5258 | Talatakely III | -21.2625 | 47.4246 | KR376059 | KR537864 |
| *G. enki* | 5259 | Talatakely III | -21.2625 | 47.4246 | KR376071 | KR537865 |
| *G. enki* | 5260 | Talatakely III | -21.2625 | 47.4246 | KR376060 |  |
| *G. enki* | 5261 | Talatakely III | -21.2625 | 47.4246 | KR376061 |  |
| *G. enki* | 5262 | Talatakely III | -21.2625 | 47.4246 | KR376068 |  |
| *G. enki* | 5263 | Talatakely III | -21.2625 | 47.4246 | KR376074 |  |
| *G. enki* | 5264 | Talatakely III | -21.2625 | 47.4246 | KR376073 |  |
| *G. enki* | 5265 | Talatakely III | -21.2625 | 47.4246 | KR376062 |  |
| *G. enki* | 5266 | Talatakely III | -21.2625 | 47.4246 | KR376063 |  |
| *G. enki* | 5267 | Talatakely III | -21.2625 | 47.4246 | KR376072 |  |
| *G. enki* | 5268 | Talatakely III | -21.2625 | 47.4246 | KR376070 |  |
| *G. enki* | 5269 | Talatakely III | -21.2625 | 47.4246 | KR376064 |  |
| *G. enki* | 5270 | Talatakely III | -21.2625 | 47.4246 | KR376065 |  |
| *G. enki* | 5271 | Talatakely III | -21.2625 | 47.4246 | KR376066 |  |
| *G. enki* | 5272 | Talatakely III | -21.2625 | 47.4246 | KR376067 |  |
| *G. boulengeri* | 5278 | Ifanadiana | -21.3019 | 47.6333 | KR376090 | KR537824 |
| *G. boulengeri* | 5279 | Ifanadiana | -21.3019 | 47.6333 | KR376091 | KR537825 |
| *G. boulengeri* | 5280 | Ifanadiana | -21.3019 | 47.6333 | KR376092 | KR537826 |
| *G. boulengeri* | 5297 | Ifanadiana | -21.3019 | 47.6333 | KR376093 |  |
| *G. boulengeri* | 5298 | Ifanadiana | -21.3019 | 47.6333 | KR376094 |  |
| *G. enki* | 5316 | Ranomafanakely | -21.4891 | 47.4891 | KR375994 | KR537846 |
| *G. enki* | 5318 | Ranomafanakely | -21.4891 | 47.4891 | KR375995 |  |
| *G. enki* | 5319 | Ranomafanakely | -21.4891 | 47.4891 | KR375996 |  |
| *G. enki* | 5320 | Ranomafanakely | -21.4891 | 47.4891 | KR375997 |  |
| *G. enki* | 5321 | Ranomafanakely | -21.4891 | 47.4891 | KR375998 |  |
| *G. enki* | 5322 | Ranomafanakely | -21.4891 | 47.4891 | KR375999 |  |
| *G. enki* | 5323 | Ranomafanakely | -21.4891 | 47.4891 | KR376000 |  |
| *G. enki* | 5324 | Station Valbio:Campsite | -21.3163 | 47.4766 | KR376032 |  |
| *G. enki* | 5326 | Station Valbio:Campsite | -21.3163 | 47.4766 | KR376033 |  |
| *G. enki* | 5327 | Station Valbio:Campsite | -21.3163 | 47.4766 | KR376034 |  |
| *G. enki* | 5328 | Station Valbio:Campsite | -21.3163 | 47.4766 | KR376035 |  |
| *G. enki* | 5329 | Station Valbio:Campsite | -21.3163 | 47.4766 | KR376031 |  |
| *G. boulengeri* | 5336 | Station Thermale | -21.4119 | 47.6983 | KR376079 | KR537830 |
| *G. enki* | 5337 | Station Thermale | -21.4119 | 47.6983 | KR376021 | KR537853 |
| *G. enki* | 5338 | Station Thermale | -21.4119 | 47.6983 | KR376022 | KR537854 |
| *G. enki* | 5339 | Kidonavo | -21.3721 | 47.4171 | KR375982 |  |
| *G. enki* | 5341 | Kidonavo | -21.3721 | 47.4171 | KR375983 |  |
| *G. enki* | 5342 | Kidonavo | -21.3721 | 47.4171 | KR375984 |  |
| *G. enki* | 5343 | Kidonavo | -21.3721 | 47.4171 | KR375985 |  |
| *G. enki* | 5344 | Kidonavo | -21.3721 | 47.4171 | KR375986 |  |
| *G. enki* | 5345 | Kidonavo | -21.3721 | 47.4171 | KR375987 |  |
| *G. enki* | 5346 | Kidonavo | -21.3721 | 47.4171 | KR375988 |  |
| *G. boulengeri* | 5349 | Ambatolahy | -21.2485 | 47.4272 | KR376080 |  |
| *G. boulengeri* | 5353 | Ambatolahy | -21.2485 | 47.4272 | KR376097 |  |
| *G. boulengeri* | 5354 | Ambatolahy | -21.2485 | 47.4272 | KR376078 |  |
| *G. enki* | 5355 | Station Valbio:Campsite | -21.3163 | 47.4766 | KR376030 |  |
| *G. enki* | 5363 | Ambohitsara/Tsitola | -21.3574 | 47.8154 | - | KR537866 |
| *G. boulengeri* | 5364 | Ambohitsara/Tsitola | -21.3574 | 47.8154 | KR376100 | KR537837 |
| *G. boulengeri* | 5365 | Ambohitsara/Tsitola | -21.3574 | 47.8154 | KR376099 | KR537838 |
| *G. enki* | 5520 | Station Valbio:Campsite | -21.3163 | 47.4766 | KR376025 |  |
| *G. enki* | 5521 | Station Valbio:Campsite | -21.3163 | 47.4766 | KR376026 |  |
| *G. enki* | 5526 | Station Valbio:Campsite | -21.3163 | 47.4766 | KR376027 |  |
| *G. enki* | 5530 | Station Valbio:Campsite | -21.3163 | 47.4766 | KR376028 |  |
| *G. enki* | 5531 | Station Valbio:Campsite | -21.3163 | 47.4766 | KR376036 |  |
| *G. enki* | 5532 | Station Valbio:Campsite | -21.3163 | 47.4766 | KR376029 |  |
| *G. enki* | 5574 | Kidanavo | -21.3721 | 47.4171 |  | KR537844 |
| *G. enki* | 5575 | Kidanavo | -21.3721 | 47.4171 | KR375979 | KR537843 |
| *G. enki* | 5576 | Kidanavo | -21.3721 | 47.4171 | KR375980 | KR537845 |
| *G. enki* | 5577 | Kidonavo | -21.3721 | 47.4171 | KR375981 | KR537812 |
| *G. boulengeri* | 5578 | Ambatolahy | -21.2485 | 47.4272 | KR376077 | KR537810 |
| *G. boulengeri* | 5579 | Ambatolahy | -21.2485 | 47.4272 | KR376076 | KR537809 |
| *G. enki* | 5580 | Ambatolahy | -21.2485 | 47.4272 | KR375971 | KR537841 |
| *G. boulengeri* | 5581 | Ambatolahy | -21.2485 | 47.4272 | KR376096 |  |
| *G. enki* | 5582 | Ambatolahy | -21.2485 | 47.4272 | KR375972 | KR537842 |
| *G. enki* | 5583 | Station Valbio | -21.3202 | 47.4522 | KR376023 | KR537855 |
| *G. enki* | 5584 | Station Valbio | -21.3202 | 47.4522 | KR376024 | KR537856 |
| *G. enki* | 5587 | Ambatolahy | -21.2485 | 47.4272 | KR375978 | KR537840 |
| *G. boulengeri* | 5588 | Ambatolahy | -21.2485 | 47.4272 | KR376075 | KR537808 |
| *G. enki* | 5595 | Ranomafanakely | -21.4891 | 47.4891 | KR376001 |  |
| *G. boulengeri* | 5860 | Ambohitsara | -21.3574 | 47.8154 | KR376095 | KR537813 |
| *G. boulengeri* | 5876 | Ambohitsara | -21.3574 | 47.8154 | KR376081 |  |
| *G. boulengeri* | 5877 | Ambohitsara | -21.3574 | 47.8154 | KR376082 | KR537811 |
| *G. boulengeri* | 5878 | Ambohitsara | -21.3574 | 47.8154 | KR376083 |  |
| *G. boulengeri* | 5879 | Ambohitsara | -21.3574 | 47.8154 | KR376084 |  |
| *G. boulengeri* | 5881 | Ambohitsara | -21.3574 | 47.8154 | KR376096 | KR537814 |
| *G. boulengeri* | 5882 | Ambohitsara | -21.3574 | 47.8154 | KR375972 |  |
| *G. boulengeri* | 5883 | Ambohitsara | -21.3574 | 47.8154 | KR376023 |  |
| *G. boulengeri* | 5884 | Ambohitsara | -21.3574 | 47.8154 | KR376024 |  |
| *G. boulengeri* | 5885 | Ambohitsara | -21.3574 | 47.8154 | KR376088 |  |
| *G. boulengeri* | 5886 | Ambohitsara | -21.3574 | 47.8154 | KR376089 |  |
| *G. boulengeri* | 8101 | Andasibe | -18.9269 | 48.4150 | KR376117 | KR537816 |
| *G. boulengeri* | 8102 | Andasibe: Torotorofotsy | -18.8666 | 48.3666 | KR376115 | KR537823 |
| *G. boulengeri* | 8103 | Andasibe: Torotorofotsy | -18.8666 | 48.3666 | KR376114 | KR537822 |
| *G. boulengeri* | 8105 | Andasibe | -18.9269 | 48.4150 | KR376113 | KR537815 |
| *G. boulengeri* | 8115 | Andasibe: Camp Prolemur | -18.9222 | 48.4929 | KR376116 | KR537821 |
| *G. boulengeri* | 8116 | Andasibe: Camp Prolemur | -18.9222 | 48.4929 | KR376118 | KR537817 |
| *G. boulengeri* | 8117 | Andasibe: Camp Prolemur | -18.9222 | 48.4929 | KR376112 | KR537818 |
| *G. boulengeri* | 8132 | Andasibe | -18.9269 | 48.4150 | KR376111 |  |
| *G. boulengeri* | 8138 | Andasibe | -18.9269 | 48.4150 | KR376110 |  |
| *G. boulengeri* | 8139 | Andasibe | -18.9269 | 48.4150 | KR376109 |  |
| *G. boulengeri* | 8140 | Andasibe | -18.9269 | 48.4150 | KR376119 |  |
| *G. enki* | 8155 | Sakaroa | -21.2679 | 47.4246 | KR376020 | KR537852 |
| *G. enki* | 8156 | Sakaroa | -21.2679 | 47.4246 | KR376019 | KR537851 |
| *G. enki* | 8157 | Sakaroa | -21.2679 | 47.4246 | KR376018 | KR537850 |
| *G. enki* | 8158 | Sakaroa | -21.2679 | 47.4246 | KR376017 |  |
| *G. enki* | 8159 | Sakaroa | -21.2679 | 47.4246 | KR376016 |  |
| *G. enki* | 8160 | Sakaroa | -21.2679 | 47.4246 | KR376013 |  |
| *G. boulengeri* | 8163 | Talatakely I | -21.2567 | 47.4236 | KR376126 | KR537831 |
| *G. boulengeri* | 8164 | Talatakely I | -21.2567 | 47.4236 | KR376128 | KR537835 |
| *G. boulengeri* | 8165 | Talatakely I | -21.2567 | 47.4236 | KR376127 | KR537836 |
| *G. enki* | 8166 | Talatakely I | -21.2567 | 47.4236 | KR376044 | KR537858 |
| *G. enki* | 8167 | Talatakely I | -21.2567 | 47.4236 | KR376043 | KR537860 |
| *G. boulengeri* | 8168 | Talatakely I | -21.2567 | 47.4236 | KR376125 |  |
| *G. boulengeri* | 8169 | Talatakely I | -21.2567 | 47.4236 | KR376123 |  |
| *G. boulengeri* | 8170 | Talatakely I | -21.2567 | 47.4236 | KR376122 | KR537834 |
| *G. boulengeri* | 8171 | Talatakely I | -21.2567 | 47.4236 | KR376121 | KR537833 |
| *G. boulengeri* | 8172 | Talatakely I | -21.2567 | 47.4236 | KR376120 | KR537832 |
| *G. boulengeri* | 8173 | Talatakely I | -21.2567 | 47.4236 | KR376108 |  |
| *G. enki* | 8177 | Talatakely I | -21.2567 | 47.4236 | KR376047 | KR537859 |
| *G. enki* | 8178 | Talatakely I | -21.2567 | 47.4236 | KR376046 |  |
| *G. enki* | 8179 | Talatakely I | -21.2567 | 47.4236 | KR376045 |  |
| *G. enki* | 8180 | Talatakely | -21.2642 | 47.4191 | KR376038 |  |
| *G. enki* | 8181 | Talatakely | -21.2642 | 47.4191 | KR376037 |  |
| *G. enki* | 8182 | Talatakely I | -21.2567 | 47.4236 | KR376040 |  |
| *G. boulengeri* | 8183 | Talatakely I | -21.2567 | 47.4236 | KR376107 |  |
| *G. enki* | 8189 | Sakaroa | -21.2679 | 47.4246 | KR376015 |  |
| *G. enki* | 8190 | Sakaroa | -21.2679 | 47.4246 | KR376014 |  |
| *G. boulengeri* | 8191 | Talatakely I | -21.2567 | 47.4236 | KR376129 |  |
| *G. boulengeri* | 8192 | Talatakely I | -21.2567 | 47.4236 | KR376130 |  |
| *G. enki* | 8193 | Ranomafana | -21.2605 | 47.4517 | KR375989 |  |
| *G. enki* | 8194 | Ranomafana | -21.2605 | 47.4517 | KR375990 |  |
| *G. enki* | 8195 | Ranomafana | -21.2605 | 47.4517 | KR375991 |  |
| *G. enki* | 8196 | Ranomafana | -21.2605 | 47.4517 | KR375992 |  |
| *G. boulengeri* | 8197 | Imaloka | -21.2421 | 47.4651 | - | KR537829 |
| *G. enki* | 8198 | Talatakely I | -21.2567 | 47.4236 | KR376042 |  |
| *G. enki* | 8199 | Talatakely I | -21.2567 | 47.4236 | KR376041 |  |
| *G. enki* | 8200 | Talatakely I | -21.2567 | 47.4236 | KR376039 |  |
| *G. boulengeri* | 8759 | Andasibe: Camp Prolemur | -18.9222 | 48.4929 | KR376104 | KR537820 |
| *G. boulengeri* | 8760 | Andasibe:Camp Prolemur | -18.8666 | 48.3666 | KR376105 |  |
| *G. boulengeri* | 8761 | Andasibe: Camp Prolemur | -18.9222 | 48.4929 | KR376103 | KR537819 |
| *G. boulengeri* | 8762 | Andasibe:Camp Prolemur | -18.8666 | 48.3666 | KR376102 |  |
| *G. boulengeri* | 8763 | Andasibe:Camp Prolemur | -18.8666 | 48.3666 | KR376101 |  |
| *G. boulengeri* | 8774 | Andasibe:Camp Prolemur | -18.8666 | 48.3666 | KR376106 |  |
| *G. boulengeri* | 11001 | Imaloka | -21.2421 | 47.4651 |  | KR537827 |
| *G. boulengeri* | 11002 | Imaloka | -21.2421 | 47.4651 |  | KR537828 |
| *G. enki* | 11003 | Talatakely I | -21.2567 | 47.4236 | KR376053 |  |
| *G. enki* | 11004 | Talatakely I | -21.2567 | 47.4236 | KR376052 |  |
| *G. enki* | 11005 | Talatakely I | -21.2567 | 47.4236 | KR376051 |  |
| *G. enki* | 11007 | Talatakely I | -21.2567 | 47.4236 | KR376050 |  |
| *G. enki* | 11008 | Talatakely I | -21.2567 | 47.4236 | KR376049 |  |
| *G. enki* | 11009 | Talatakely I | -21.2567 | 47.4236 | KR376048 |  |
